# Marker development using SLAF-seq and whole-genome shotgun strategy to fine-map the semi-dwarf gene *ari-e* in barley

**DOI:** 10.1186/s12864-016-3247-4

**Published:** 2016-11-11

**Authors:** Qiaojun Jia, Cong Tan, Junmei Wang, Xiao-Qi Zhang, Jinghuan Zhu, Hao Luo, Jianming Yang, Sharon Westcott, Sue Broughton, David Moody, Chengdao Li

**Affiliations:** 1 College of Life Sciences, Zhejiang Sci-Tech University, Hangzhou, 310018 China; 2 Key Laboratory of Plant Secondary Metabolism and Regulation of Zhejiang Province, Hangzhou, 310018 China; 3 Western Barley Genetics Alliance, Murdoch University, Murdoch, WA 6150 Australia; 4 Institute of Crop and Nuclear Technology Utilization, Zhejiang Academy of Agricultural Sciences, Hangzhou, 310021 China; 5 Department of Agriculture and Food Government of Western Australia, South Perth, WA 6155 Australia; 6 InterGrain Pty Ltd, 19 Ambitious Link, Bibra Lake, WA 6163 Australia

**Keywords:** Barley, Semi-dwarf, SLAF, Whole-genome sequence, Fine-map

## Abstract

**Background:**

Barley semi-dwarf genes have been extensively explored and widely used in barley breeding programs. The semi-dwarf gene *ari-e* from Golden Promise is an important gene associated with some agronomic traits and salt tolerance. While *ari-e* has been mapped on barley chromosome 5H using traditional markers and next-generation sequencing technologies, it has not yet been finely located on this chromosome.

**Results:**

We integrated two methods to develop molecular markers for fine-mapping the semi-dwarf gene *ari-e*: (1) specific-length amplified fragment sequencing (SLAF-seq) with bulked segregant analysis (BSA) to develop SNP markers, and (2) the whole-genome shotgun sequence to develop InDels. Both SNP and InDel markers were developed in the target region and used for fine-mapping the *ari-e* gene. Linkage analysis showed that *ari-e* co-segregated with marker InDel-17 and was delimited by two markers (InDel-16 and DGSNP21) spanning 6.8 cM in the doubled haploid (DH) Dash × VB9104 population. The genetic position of *ari-e* was further confirmed in the Hindmarsh × W1 DH population which was located between InDel-7 and InDel-17. As a result, the overlapping region of the two mapping populations flanked by InDel-16 and InDel-17 was defined as the candidate region spanning 0.58 Mb on the POPSEQ physical map.

**Conclusions:**

The current study demonstrated the SLAF-seq for SNP discovery and whole-genome shotgun sequencing for InDel development as an efficient approach to map complex genomic region for isolation of functional gene. The *ari-e* gene was fine mapped from 10 Mb to 0.58 Mb interval.

**Electronic supplementary material:**

The online version of this article (doi:10.1186/s12864-016-3247-4) contains supplementary material, which is available to authorized users.

## Background

Semi-dwarf genes were crucial in the green revolution because they conferred semi-dwarfness, which reduced lodging and increased crop yields [1–3]. Most of the green revolution semi-dwarf varieties resulted from a gene mutation in the gibberellic acid (GA) pathways, e.g. rice *sd1* gene, wheat DELLA genes and maize *d8* [1–5]. For barley, the world's fourth leading cereal crop, semi-dwarf genes such as *sdw1/denso*, *uzu1* and *ari-e* have been extensively explored and widely accepted in barley breeding programs in different regions. Barley *sdw1/denso* varieties carry a mutation in GA 20 oxidase-2, a GA biosynthesis gene that is homologous to rice *sd1* [6–9], which has been used to develop short-stature cultivars in western USA, Canada, Australia and European countries [10–14]. The *uzu1* mutant had a significantly reduced response to brassinolide resulting from a missense mutation in the BR receptor gene *HvBRI1* and has been widely used in East Asia [14–17]. The *ari-e* gene from Golden Promise is a commonly-used semi-dwarf gene in spring barley in Europe, especially Scotland. A new barley variety with the *ari-e* gene, Hindmarsh, has been grown in nearly half of Australia. Besides the magnitude of the effect of this gene on reducing plant height, *ari-e* has significant effects on some agronomic traits, such as grain size, grain composition, malting quality and yield [18, 19]. Golden Promise is a model cultivar for barley genetic transformation [20, 21]. Recently, some studies have reported that *ari-e* is the only semi-dwarf gene to improve salt tolerance [19, 22–24]. Genetic analysis showed that *ari-e* was a recessive semi-dwarf gene and was mapped on barley chromosome 5H as a quality character or quantitative trait locus (QTL) using traditional markers and next-generation sequencing technologies [19, 22, 23, 25, 26]. However, the *ari-e* gene is yet to be identified.

Map-based cloning is the most promising approach for molecular isolation of target genes. The first map-based cloning gene in barley was the powdery mildew resistance gene *mlo* [27]. A growing number of genes have been isolated from barley based on genetic information. Eight genes are involved in disease resistance against powdery mildew (*mlo*, *Rar1*, *mla* and *Ror2*) [27–30], stem rust (*Rpg1* and *Rpg4/Rpg5*) [31, 32], barley yellow mosaic virus (*rym4/rym5*) [33] and leaf strip (*Rdg2a*) [34]. Besides resistance genes, some of the genes identified by map-based cloning contribute to complex agronomic traits such as photoperiod sensitivity (*Ppd-H1*) [35], vernalization requirement (*Vrn3*) [36], row type (*Vrs1*) [37], tolerance to boron toxicity (*Bot1*) [38], covered/naked caryopsis (*nud*) [39], anthocyanin pigmentation (*ant2*) [40], floret closing (*Cly1*) [41], awn length (*lsk2*) [42], increased number of internodes (*mnd*) [43], leaf color variation (*HvSGRA*) [44] and branch development (*Cul4*) [45]. A larger number of molecular markers are the basis of map-based cloning for genes related to complex agronomic traits. There are thousands of molecular markers (RFLP, AFLP, SSR, STS, SNP and DArT) available for barley genomes (http://wheat.pw.usda.gov/GG2/index.shtml, http://bioinf.scri.ac.uk/barley_snpdb/index.html, http://www.diversityarrays.com/), allowing an efficient and rapid localization of genes at low resolution. Further narrowing of the target region and distinguishing which gene is responsible for the interested traits is limited by the size of the segregating population and molecular marker density.

Recently, the ongoing revolution in sequencing techniques has significantly reduced costs and advanced technologies to provide new strategies for discovering thousands of markers. Next-generation sequencing (NGS) facilitates the rapid identification of genomic variants for marker development in most species. For example, NGS identified 416,856 markers in wheat [46] and 11,805 SNP markers in cotton [47]. In barley, 1,391 high confidence SNPs generated from genotyping by sequencing (GBS) were used to construct a linkage map of doubled haploid (DH) Golden Promise × Morex (GPMx) RILs population [26]. Aligning genomic DNA sequences between two sequenced barley varieties, Morex and Barke, identified 436,640 InDel markers [48].

Bulked segregant analysis (BSA) combined with NGS technologies offers new strategies for marker discovery associated with genes/QTLs. Specific-length amplified fragment sequencing (SLAF-seq) is considered an efficient and high-resolution genotyping method [49], which has identified major QTLs in maize [50], soybean [51], cucumber [52], rice [53] and candidate gene isolation in cucumber [54] and barley [44]. In this study, traditional marker assays and the combination of BSA with SLAF-seq were used to detect a genomic region in barley harboring the semi-dwarf gene *ari-e*. SNPs and InDel markers were developed by SLAF-seq and whole-genome shotgun sequencing technology, respectively, to fine-map *ari-e*. The current study exemplifies the use of SLAF-seq for SNP discovery and whole-genome shotgun sequencing for InDel development in the target region, which may pave the way for map-based cloning of the *ari-e* gene and unraveling the molecular mechanisms of the semi-dwarf phenotype.

## Results

### Classical mapping of the *ari-e* gene

**Fig. 1:**
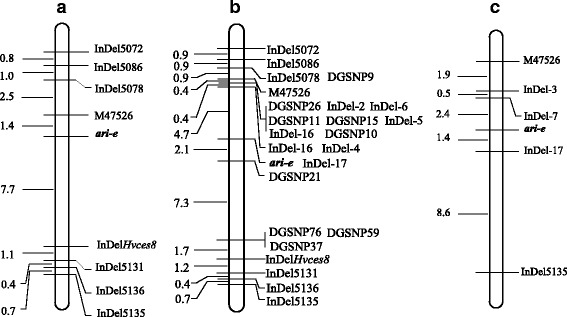
Genetic map of the *ari-e* gene on barley chromosome 5H. **a** Primary mapping of barley semi-dwarf gene *ari-e* in the Dash × VB9104 DH population. **b** Mapping developed markers in the Dash × VB9104 DH population. **c** Mapping *ari-e* in the Hindmarsh × W1 DH population

According to previous linkage mapping, *ari-e* is located on chromosome 5H between SSR marker Bmag337 and Bmag357 in Derkado × B83-12/21/5 DH population [19]. Twenty-nine InDel markers in the target region developed by Zhou et al. [48] were tested in the parents of Dash and VB9104. Of these, six markers (InDel5072, InDel5078, InDel5086, InDel5131, InDel5135 and InDel5136) showed polymorphism. Based on the current barley assembly information, *Hvces8* (*MLOC_68431*), which is homologous to rice cellulose synthase catalytic subunit genes 9 (*OsCesA9*), is also in the target region. After sequencing the *Hvces8* gene in Dash and VB9104, there was 6 bp (GCATCG) insertion in its 5’untranslated region in VB9104. Therefore, the InDel marker of *Hvces8* was designed. Two SSR markers were developed from the sequence of Morex_contig47526, one of which was polymorphic between the parents. As a result, seven InDel markers and one SSR marker (Table 1) were used to genotype 119 individuals from the Dash × VB9104 DH population and to construct the linkage map (Fig. 1a). The *ari-e* gene was mapped as a phenotypic marker and was located to a region on chromosome 5H, delimited by M47526 (1.4 cM) and InDel*Hvces8* (7.7 cM). The flanking markers (M47526 and InDel*Hvces8*) interval corresponded to 15.6 Mb on the International Barley Sequencing Consortium (IBSC) physical map.

**Table 1:** PCR markers used to classical mapping

Marker name	Forward sequence	Reverse sequence	Size	POPSEQ Phyiscal Position
InDel5086	TTGCGAACACGGACTCTGAG	TAAATTGCGGCCAAGGGACA	139 bp	Chr 5H 392,878,714
InDel5072	TGGTCCAGAACACGGATACC	AAGAGTTGGCGCCAGATGAG	142 bp	Chr un 71,938,413
InDel5078	GGCGAGGGAGGAGAAGAGTA	GTAGACCTCCCCTCCCTCTC	112 bp	Chr 5H 394,298,209
InDel5131	GGGAACGTGAGGCCTAATGT	CCTCTTCTCCAAGTGACGGG	115 bp	Chr 5H 498,813,832
InDel5135	GTGCCGTGAAACACATGCAT	AAGCAACTAACCGCGATTGC	127 bp	Chr 5H 499,924,212
InDel5136	GAGGGGTCAGACTGATGTGC	AGGTCGATCCTCATTGCCAC	113 bp	Chr 5H 499,918,835
InDel*Hvces8*	GGCGACGGCAACAACACCC	GAGAGCTGGATGGAGAGGGAG	120 bp	Chr 5H 495,938,660
M47526	GTTTCAGGTACAGAAGCCAACG	AGATCAGGAAGCGGACCAACC	178 bp	Chr 5H 478,393,185

### Analysis of SLAF-seq data and SLAF tags

A total of 63,634,776 fragments were procured, each with read lengths of ~100 bp (Table 2). Most of the bases (93.51 %) were high quality, with quality scores of at least 30 (Table 2). The SLAF numbers were 190,519 for Dash and 217,281 for VB9104. The average depth of the SLAF markers was 19.99-fold in Dash, 15.51-fold in VB9104, 30.19-fold in the semi-dwarf pool and 30.34-fold in the highpool. According to the results of SLAF positioning on the barley genome, 319,656 SLAF tags were anchored; the SLAF numbers and chromosome positions are shown in Table 3.

**Table 2:** Statistic results of sequencing data for both parents and bulked DNA pools

Sample	Total reads	GC %	Q30 %	SLAF number
Dash	10,661,428	43.52	93.70	190,519
VB9104	10,366,368	43.67	93.73	217,281
Semi-dwarf pool	20,586,641	43.62	93.03	236,077
High pool	22,020,339	43.42	93.59	248,167
Total	63,634,776	——	——	892,044

**Table 3:** Number distribution of SLAF tags, SNP markers and polymorphic SNP on each chromosome

Chromosome	SLAF number	All SNP	Polymorphic SNP
Chr 1H	34,982	49,781	6,536
Chr 2H	50,035	95,116	12,153
Chr 3H	46,287	71,052	9,292
Chr 4H	45,177	49,973	9,197
Chr 5H	44,446	61,384	8,938
Chr 6H	41,097	65,269	12,652
Chr7H	47,353	78,746	15,893
Chr unknown	10,297	44,623	7,623
Total	319,656	515,944	82,284

### Polymorphic SNPs and association analysis

**Fig. 2:**
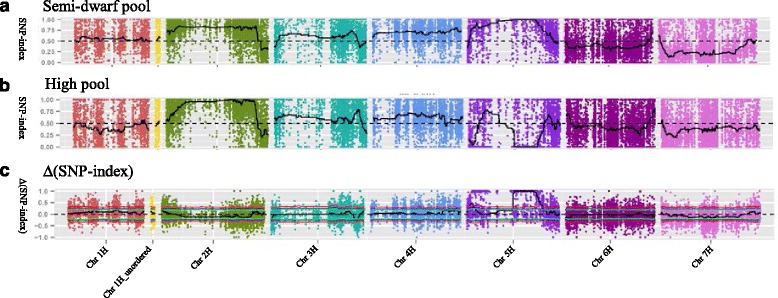
Indentification of the semi-dwarf gene *ari-e* related candidate regions through SNP-index association analysis. SNP-index graphs of Semi-dwarf pool (**a**) and High pool (**b**) and Δ(SNP-index) (**c**) graph from SLAF-seq analysis. X-axis represents the position of seven chromosomes and Y-axis represents the SNP-index

After aligning the sequence data to the barley reference, SNPs that differed from the reference sequence were identified. At the stage of SNP calling, each SNP supported by at least four reads in semi-dwarf or high pools were filtered out and 193,626 SNP were detected. Of these SNPs, 82,284 SNPs were polymorphic between Dash and VB9104 and were ultimately selected for association analysis. The statistics of marker numbers on each chromosome according to the positioning result are shown in Table 3. The SNP-index was calculated for each SNP. An average SNP-index of SNPs was calculated with 200 SNP-indexes located in a given genomic interval. SNP-index graphs were generated for the semi-dwarf (Fig. 2a) and high (Fig. 2b) pools by plotting the average SNP-index against the position of each sliding window in the barley genome assembly. After combining the SNP-index information into the semi-dwarf and highpools, the ∆(SNP-index) was calculated and plotted against the genome positions (Fig. 2c). According to the IBSC physical map, the candidate region on chromosome 5H between 279,152, 772 and 460,183,011 was obtained with 99 % confidence.

### Develop SNP markers based on SLAF-seq

After aligning the marker sequence to the barley physical map, the preliminary mapping region of *ari-e*, defined by M47526 and InDel*Hvces8*, corresponded to 15.6 Mb (from 387,773,791 to 403,357,465 bp on chromosome 5H of barley IBSC assembly). Combining the preliminary mapping and SLAF-seq technology suggested that the overlap of both results was delimited by M47526 and InDel*Hvces8* and was the candidate region of the semi-dwarf gene *ari-e*. To further evaluate the accuracy of SLAF genotyping data, SNPs which showed polymorphism between semi-dwarf and high pools, and were not segregated in each pool, were selected across the entire candidate region. Selected SNPs with one genotype derived from Dash and the other from VB9104 were designated as potential markers. Ultimately, eight SNPs and one InDel (DGSNP76) were identified after performing independent traditional Sanger sequencing in Dash and VB9104. Their primer sequences and physical map positions are shown in Table 4.

**Table 4:** PCR primers, product size and physical position designed from SLAF-seq strategy

Marker name	Forward sequence	Reverse sequence	Size	IBSC Physical Position	POPSEQ Phyiscal Position
DGSNP9	GGCATAACGGTAACCCAATG	TCAAATAGCCGTGTCCATGA	232 bp	Chr 5H 387,821,508	Chr 5H 451,386,904
DGSNP10	GTTGGCCCAAGATTTATCCC	CGACCCTGATCAAGAACACA	224 bp	Chr 5H 389,006,171	Chr 5H 487,359,370
DGSNP11	CTCATGACCAACGCTTTCAA	CTGTCGGTCAAGGACACAAA	132 bp	Chr 5H 389,903,941	Chr 5H 483,326,936
DGSNP15	ATTATGTGTTCCTGGGCCTG	CAGAAGGCAATGGATGATGA	113 bp	Chr 5H 393,057,839	Chr 5H 486,659,431
DGSNP21	ACATTCATGTTTCCCCGTGT	GGAAAGGACTAGTAGGGCCG	126 bp	Chr 5H 396,709,139	Chr 5H 490,942,368
DGSNP26	ACCCAATTCTCATAGGCACG	GAGAGGGGGAACACGTACAA	158 bp	Chr 5H 399,664,776	Chr 5H 480,758,192
DGSNP37	TCCCTTGCCAGAAAAACATC	TACGCAGCCTATACCATCCC	261 bp	Chr 5H 399,776,063	Chr 5H 494,670,447
DGSNP59	TTGGGATCTTTATGGCAAGC	TATTCCGGTACCTGCACCTC	238 bp	Chr 5H 400,302,818	Chr 5H 494,361,321
DGSNP76	CTTTCTTTGTAGTTGTACGCAT	TGCGTTAAAAGGCCTAAACA	144 bp	Chr 5H 400,112,902	Chr 5H 492,409,362

### Develop InDel markers by whole-genome shotgun strategy

There were 2,029,068,470 clean reads with a read length of at least 100 bp after resequencing the barley cultivar, Hindmarsh. Moreover, 98.64 % (2,001,398,858 reads) were mapped to the recently released barley reference genome assembly by population sequencing (POPSEQ) methodology [55]. About 5.7 million variations, including 412,597 small InDels, were identified in the whole genome. There were 50, 767 InDels on chromosome 5H. According to the POPSEQ assembly, the *ari-e* candidate region between M47526 and InDel*Hvces8* spans a total sequence length of approximately 17.5 Mb (478,393,185 to 495,938,660). Therefore, 17 InDels were selected to design primers in the *ari-e* targeted region (Table 5). The size comparison of PCR products on PAGE gels revealed insertion or deletion polymorphisms between Dash and VB9104. Ultimately, seven InDels (InDel-2, −4, −5, −6, −13, −16 and −17) showed size polymorphism between Dash and VB9104.

**Table 5:** PCR primers, product size and their physical position designed from whole-genome shotgun strategy

Marker name	Forward sequence	Reverse sequence	Size	POPSEQ Phyiscal Position
InDel-1	TTATCCCCACTTACAGCCCG	GGTAGTACCGCTAGGGGCA	80 bp	Chr 5H 487,228,854
InDel-2	GTTTAGCAGGGAGGCTCGAA	GAATATCTGGCCATGCATGCA	130 bp	Chr 5H 482,479,881
InDel-3	GCGAAACCTTCCTTGCTTCC	TTTGACAAGGTTGGTCTCAAAAA	125 bp	Chr 5H 481,610,815
InDel-4	AGCCAACGGTAGGTACATGC	GCTGCTGCTAGGTCAGAAAGA	122 bp	Chr 5H 487,890,216
InDel-5	CACCCATATTTGTGCATAGGC	TGAGGTTGTGGATTGACGAACA	120 bp	Chr 5H 486,795,826
InDel-6	TCAGGTCCCTTCTTCAGTGA	AGTGCCAGCCAAATGCAGTA	106 bp	Chr 5H 482,690,738
InDel-7	GTTTAAAGCCGGTGAGCGTG	ACGTTGTTATTTATACAACACAGGG	83 bp	Chr 5H 482,623,538
InDel-8	AGGAATTAACAGGACAAATTTAGCA	TCATGGTGAAACATGGTGAATCT	107 bp	Chr 5H 481,623,681
InDel-9	GGTACTACCGCTCGCGAG	GCCAGCAGTAGTACCGCTAG	101 bp	Chr 5H 481,632,966
InDel-10	CAATTGTTCAGGCAAAAATTCA	CTCTCGAGGAAAGCAGGTAATG	139 bp	Chr 5H 481,342,502
InDel-11	GATACTGAAATCTTGCCCATGC	TCAATTGCTTTGTTTGTGGAAC	180 bp	Chr 5H 481,701,251
InDel-12	TGTTGCCCTCTGGTATGAATAG	AATTTCAGTGTCTGAACTATGGG	136 bp	Chr 5H 482,525,089
InDel-13	CCTTCTTCGTCTAGCACCCATA	TGCTCGTACTGAGGTTTCTTGA	175 bp	Chr 5H 486,795,676
InDel-14	ACAGTAGCGCCAGTAATTGTGT	AGTTTCCAGAGTATCACTGCCA	180 bp	Chr 5H 487,123,960
InDel-15	TGTGTGGCAGTTTCTTTAATGG	TGAAGCTTTGTTTTACTGACGG	176 bp	Chr 5H 487,521,136
InDel-16	AACAAGACCTGGAGAGACAAGC	TGCTGCTAGGTCAGAAAGAAAA	138 bp	Chr 5H 487,890,066
InDel-17	CTCCTACCACCCTTTTCACCC	TACTGCAAGAAATCGTACCACC	154 bp	Chr 5H 488,465,184

### Mapping the developed markers in the Dash × VB9104 DH population

Fine-mapping with all of the polymorphic markers was performed in 119 Dash × VB9104 DH individuals (including lines from the semi-dwarf and high pools). After independent traditional Sanger sequencing, the markers derived from different SLAF sequences correlated well with the SLAF-seq genotyping information, suggesting that the markers mined from Dash, VB9104, semi-dwarf and high pools are reliable. InDel markers have unique amplicons and can be distinguished easily on 6 % polyacrylamide gel in the population. Linkage analysis of 119 Dash × VB9104 DH individuals showed that all markers were assigned to the target regions, and 15 of 16 markers were mapped between marker M47526 and InDel*Hvces8* intervals. Moreover, the InDel-17 marker co-segregated with *ari-e*. Accordingly, *ari-e* was delimited to a 6.8 cM region flanked by InDel-16 and DGSNP21 (Fig. 1b). The mapping results also confirmed the genotyping accuracy of SLAF-seq and the efficiency of marker discovery using whole-genome shotgun strategy.

### Validation of markers on Hindmarsh × W1 DH population

To confirm the *ari-e* position, we conducted linkage analysis with 340 DH plants from the Hindmarsh × W1 cross. Five polymorphic markers including M47526, InDel-3, InDel-7, InDel-17 and InDel 5135 were applied to the segregating population for *ari-e* mapping. Linkage analysis identified the *ari-e* semi-dwarf gene delimited by two InDel markers InDel-7 (2.4 cM) and InDel-17 (1.4 cM), which were physically located in the region of 482,623,538 to 488,465,184 bp (POPSEQ) on chromosome 5H (Fig. 1c). The mapping position of *ari-e* in the Hindmarsh × W1 DH population was consistent with that in the Dash × VB9104 DH population and the overlapped region identified by the two populations was considered the *ari-e* candidate region, which allowed further narrowing of the *ari-e* locus to an 0.58 Mb interval between InDel-16 (487,890,066) and InDel-17 (488,465,184) on the barley POPSEQ physical map.

### Analysis of the candidate region and gene annotation

In this 0.58 Mb region, only five genes were predicted according to the barley POPSEQ physical map, and the gene annotations are presented in Table 6. Mazzucotelli et al. [56] reported that E3 ubiquitin ligases play a major role in protein degradation and are involved in plant growth and development. Therefore, *MLOC_66038* caught our attention, based on the gene annotation of barley which acts as the E3 ubiquitin-protein ligase, and seemed most likely to be responsible for the semi-dwarf phenotype. Sequence analyses of Hindmarsh, however, showed no difference in *MLOC_66038* compared with the reference genome Morex. Alignment of the nucleotide sequences in the other four genes also revealed no variation between Hindmarsh and Morex.

**Table 6:** Predicted genes in the candidate region

Gene	Annotation	POPSEQ Phyiscal Position
*HvLOC4347149*	Putative protease Do-like 14 isoform *X*2	Chr 5H 487,897,795–487,907,005
*MLOC_15960*	Uncharacterized protein LOC100825869	Chr 5H 488,009,510–488,010,767
*MLOC_66038*	E3 ubiquitin-protein ligase RNF14-like	Chr 5H 488,351,650–488,352,677
*MLOC_72534*	F-box/Kelch-repeat protein SKIP11	Chr 5H 488,362,230–488,366,885
*HvLOC100842002*	Glycine tRNA synthetase 2, chloroplastic/mitochondrial	Chr 5H 488,367,655–488,371,640

Genetic variants of the target region were also identified and are listed in Additional file 1: Table S1. Most of the variants were in the intergenic region, with only a few located in upstream and downstream regions of the predicted genes. Such variants were at least 2.0 Kb and 1.5 Kb away from the upstream and downstream regions of the predicted genes, respectively (Additional file 1: Table S1). To test if the variants affect the expression pattern of the predicted genes, we designed five primer pairs (Additional file 2: Table S2) for qRT-PCR analysis in VB9104, Dash and Golden Promise. According to the qRT-PCR results, the relative expression levels of *MLOC_15960*, *MLOC_72534* and *HvLOC100842002* had not significantly changed in the three varieties (Additional file 3: Figure S1). However, *HvLOC4347149* and *MLOC_66038* exhibited higher expression levels in Golden Promise and lower expression levels in Dash compared with the VB9104 (Additional file 3: Figure S1), which indicates that both genes exhibit no consistent expression with *ari-e*. It seems that none of the five predicted genes was responsible for the *ari-e* semi-dwarf phenotype based on our genetic map and the recent release of the barley draft genome assembly.

## Discussion

Traditional methods for developing markers are tedious, time-consuming and expensive [57]. Limited number of markers in the barley genomic region is a major obstacle in the fine-mapping of barley genes, which has encouraged the development of new marker systems that have a greater degree of polymorphism. SLAF-seq is a relatively new experimental method which is considered an efficient and high-resolution strategy for large-scale genotyping using an enhanced reduced representation library (RRL) sequencing method [49]. It is an effective, low-cost technology for constructing high-density linkage maps, which has successfully facilitated the identification of major QTLs related to complex traits in plants [50, 51, 53]. Using the SLAF-seq approach, an SNP-based saturated genetic map was constructed and a major fruit length QTL, which explained 44.6 % of the phenotypic variance in cucumber, was detected [52]. Combining SLAF-seq with BSA identified chromosome regions related to 1000 grain weight in rice and the gene that control inflorescence meristem function in maize [50, 53]. Xu et al. [54] reported that fruit flesh thickness in cucumber was fine-mapped by two SLAF markers in the 0.19 Mb genomic region. After anchoring the markers to the cucumber 9930 reference genome, 20 genes were predicted, and *Csa2M058670.1* was identified as a good candidate gene due to its high expression level in thick fruit flesh varieties. In some cases, the interval identified by SLAF-seq is too large to isolate the target gene. The mapping region of barley *HvSGRA* (Stage Green-Revertible Albino Gene) defined by SSR and SLAF-seq, was insufficient to pin the candidate gene in barley [44]. Therefore, new markers based on the barley physical map were designed to further fine-mapping and its candidate gene was isolated according to its annotation and a nonsense mutation. In the present study, 319,656 SLAF tags were developed by high-throughput sequencing, with 82,284 SNPs identified (Table 3). Associated analysis with the SNP-index obtained semi-dwarf gene *ari-e* related candidate regions on chromosome 5H with a size of 181.03 Mb (Fig. 2). The large candidate region of *ari-e* by SLAF-seq was possibly due to the low recombinants between semi-dwarf and high pools in the target region. After combining with primary mapping, the SNP markers converted from polymorphic SLAFs provide useful data for restricting the candidate-associated regions into 7.2 cM interval, delimiting an approximate 3.6 Mb region in 119 Dash × VB9104 DH lines. Therefore, SLAF-seq is a highly-efficient strategy for mapping the candidate gene, but further work needs to be done to refine the target interval to isolate the candidate, especially in barley with such a large haploid genome.

InDel markers are a gel-based molecular marker which has played a major role in genetic studies and QTL mapping in rice and *Arabidopsis* [58–60]. The recent release of barley draft assembly sequences provides a new approach to finding insertion/deletion polymorphisms in barley varieties at the DNA level. A total of 436,640 InDels were identified between two sequenced barley varieties, Morex and Barke, based on genome-wide alignment, and 1,140 InDel markers were integrated with the barley consensus map [48]. Taking the same approach, we developed InDel markers in the target region; the newly-designed polymorphic InDel markers were linked with *ari-e* (Fig. 1b, c). Of these, InDel-17 marker co-segregated with *ari-e* and we narrowed the *ari-e* gene to the region flanked by InDel-16 and DGSNP21 in the Dash × VB9104 DH population (Fig. 1b). Most of the developed markers were co-segregated, possibly due to the small population size. Furthermore, we validated the mined markers to another mapping population (Hindmarsh × W1 DH population), and the *ari-e* gene was flanked by InDel-7 and InDel-17 (Fig. 1c). Combining both mapping results, *ari-e* can be narrowed between InDel-16 and InDel-17. As a result, alignment of the whole-genome shotgun sequence with the released barley genome sequence enabled the discovery of a large number of DNA markers. This study is an example of the efficient exploitation of targeted InDel markers developed by the whole-genome shotgun strategy in barley. Different alleles at the *ari-e* locus such as such as *ari-e.1*, *ari-e.119*, *ari-e.156* and *ari-e.228* described by Kucera et al. [61], were induced by ionizing radiations (neutrons and X-ray) and by chemical mutagens (propyl methanesulfonate and N-methyl-N-nitrosourea). Allelism to the *ari-e* mutant showed that *ari-e.1* and Golden Promise mutant are alleles [62]. The variety Golden Promise (a gamma-ray mutant of the variety Maythorpe) with *ari-e* is famous for its desirable agronomic traits such as earliness, short stiff straw, reduced awn length and better salt tolerance compared with other semi-dwarf varieties [23, 63]. Considering its importance to barley breeding, *ari-e* has been genetically characterized in numerous genetic backgrounds. Thomas et al. [25] used the phenotypic markers in both F2 populations, and a doubled monoploid population to genetically map the *ari-e* locus to chromosome 5H of barley near the centromere. In the AFLP and SSR marker map constructed for the Derkado × B83-12/21/5 DH population, the *ari-e* gene was flanked by SSR marker Bmag337 (3 cM) and Bmag357 (7 cM) [19]. After genotyping with the 1,536-SNP Illumina GoldenGate oligonucleotide pool assay in a three-way barley cross, Malosetti et al. [64] identified that *ari-e* was close to SNP marker 2_1239 which has been included in the barley integrated map of Aghnoum et al. [65] in 5H bin 6. Based on Illumina’s oligo pool assays (OPAs) (the same SNP genotyping), *ari-e* is associated with SNP markers 1_1198 to 2_0449 in 5H bins 2 to 9 in BW043, which resulted from the introgression *ari-e* into the background of cv. ‘Bowman’ [66]. All these results confirm that barley 5H bin 6 encompasses the major semi-dwarf gene *ari-e*. Using GBS data of GPMx RILs population, *ari-e* is closely linked with GBS marker MR_47526P1793R57, which is flanked by MR_335403P1239R45 and MR_1560792P1192F41 [26]. These markers defined a 7.2 cM interval on the GPMx GBS map. According to the barley IBSC physical map, the interval defined at least 46 Mb and contained an estimated 397 genes [26]. The same interval spanned a total sequence length of approximately 10 Mb on the barley POPSEQ map [26]. The authors explained that both the relatively low recombination and lack of detected polymorphism resulted in the low-resolution map in the *ari-e* region of the GPMx RILs population. In our initial mapping, *ari-e* was defined at a 9.1 cM interval, which corresponds to 17.5 Mb on the barley POPSEQ physical map. Although the genetic distance was similar, the physical map interval of *ari-e* defined by M47526 and InDel*Hvces8* in the Dash × VB9104 DH population was larger than that delimited by MR_335403P1239R45 and MR_1560792P1192F41 in the GPMx RILs population. The target region decreased to 6.8 cM covering the physical map from 487,890,066 bp to 490,942,368 bp intervals in the Dash × VB9104 DH population. After we had validated the polymorphic markers to the Hindmarsh × W1 DH population, *ari-e* was flanked by InDel-7 and InDel-17 and the genomics DNA interval of 482,623,538 to 488,465,184 bp on chromosome 5H. Based on the barley physical map, its physical position and combining the mapping results of both populations, the candidate region of *ari-e* declined to an interval of 0.58 Mb, which represents significant progress toward identifying the functional gene compared to previous studies [26].

Ellis et al. [19] reported that recessive dwarf mutants in barley indicate a loss of function. It was confirmed in both barley recessive semi-dwarf mutants, *uzu1* and *sdw1/denso*, which resulted from the loss of function of the BR receptor gene *HvBRI1* and gibberellin biosynthesis gene *HvGA20ox*_*2*_, respectively [8, 15]. Genetic analysis showed that *ari-e* mutants also carried a recessive allele [25]. We deduced that the semi-dwarfism of *ari-e* was possibly due to the mutation of one of the functional genes. According to the most current POPSEQ barley map [55], there are five predicted genes in the candidate region (Table 6). Considering the semi-dwarf phenotype of *ari-e* and the gene annotation of the five genes, *MLOC_66038* predicted as an E3 ubiquitin-protein ligase may be a candidate gene for *ari-e*. E3 ubiquitin ligase members are involved in the regulation of some biological processes including hormonal control of vegetative growth, plant reproduction, light response, biotic and abiotic stress tolerance, and DNA repair [56]. For example, both *Arabidopsis sly1* and rice *gid2* encode the F-box subunit of SCF E3 ubiquitin ligase that regulates GA responses and results in the dwarf phenotype [67, 68]. Unfortunately, no nucleotide sequence variants of *MLOC_66038* or the other four genes were present between the *ari-e* variety Hindmarsh and the reference variety Morex.

In barley, semi-dwarf gene *sdw1/denso* affects not only plant height but also multiple agronomic traits, because *sdw1/denso* encodes GA 20-oxidase and is involved in gibberellin biosynthesis [6–8]. Therefore, *sdw1/denso* is expressed all of the tissues and affects many developmental processes through GA level [7, 8]. *ari-e* also reduces plant height and is related with lots of agronomic traits, such as erect juvenile growth habit, earliness, compact inflorescene, short awns, small seed size and cell size reduction in leaf blades [19, 24, 25]. This indicated that *ari-e* may be involved in different developmental processes. Furthermore, reduced expression of *HvGA20ox*_*2*_ was identified in various organs in *denso* semi-dwarf mutant compared with tall variety [7]. Rieu et al. [69] reported that *AtGA20ox*_*1*_ (also named as semi-dwarf gene *ga5*) was showed different expression in different tissues, and at different developmental stages. The difference of plant height between *ari-e* semi-dwarf and tall varieties reaches the maximum at the heading time and the uniform leaf samples can easily be collected. Therefore, leaves of VB9104, Dash and Golden Promise were collected to detected relative expression levels of all predicted genes at the heading stage. But expression levels of the five genes were not consistent with *ari-e*. The unexpected results might be due to the limited resolution of the current barley physical map while the non-coding region may play a key role in inducing semi-dwarfness. Owing to the large size of the barley genome (5.1 Gb) and the high repeat content (80 %), the current physical map comprises 9,265 contigs with a cumulative size of 4.9 Gb representing 96 % of the physical length of the barley genome [70]. Chromosomal assignments between the POPSEQ and IBSC maps agree in 97.6 % of the cases and discordant contig placements mostly occurred in the genetic centromere due to the severely reduced recombination frequency [55, 68]. Moreover, most of the map-based cloned barley genes have been located in the distal regions of the chromosome. Considering that *ari-e* occurred near the centromere of barley 5H [25], the repetitive nature of the barley genome [55, 70, 71] and limited resolution of the barley physical map, there may be other unanchored candidate genes for the *ari-e* semi-dwarf gene.

In summary, next-generation sequencing combined with the current barley physical map served as a hub for marker development in the desired region and the candidate gene will be isolated with the ongoing construction of the high-resolution barley physical map.

## Conclusions

Traditional marker assays and SALF-seq in conjunction with BSA were conducted to map the semi-dwarf gene *ari-e*. SNPs and InDel markers were developed by SLAF-seq and whole-genome shotgun sequencing technology, respectively, to fine-map *ari-e*. The *ari-e* gene was mapped between two developed markers InDel-16 and InDel-17 with an interval of 0.58 Mb. The current study exemplifies the use of SLAF-seq for SNP discovery and whole-genome shotgun sequencing for InDel development in the target region, which may pave the way for map-based cloning of the *ari-e* gene and unraveling the molecular mechanisms of the semi-dwarf phenotype.

## Methods

### Plant materials

Two mapping populations and their parents were used for the genetic analysis and molecular mapping of the *ari-e*. The first population was comprised of 119 DH lines produced from a cross between Dash and VB9101, and the second DH population was derived from a cross of Hindmarsh × W1 with 340 lines. Hindmarsh with semi-dwarf phenotype, was selected from the Dash × VB9409 cross and used in whole-genome shotgun sequencing for marker development. Dash is a short, stiff straw cultivar with *ari-e* derived from Golden Promise. Thus, the semi-dwarf phenotype of Hindmarsh can be traced back to Golden Promise. Both VB9104 and W1 are mid-tall accessions. Leaves of VB9104, Dash and Golden Promise were harvested at the heading stage for RNA extraction. Each variety was collected two samples with four individual lines.

### DNA preparation

Young healthy leaves from the parents and DH individuals were collected, frozen in liquid nitrogen and used for DNA extraction. Genomic DNA was extracted from each plant using the cetyl-trimethyl-ammonium bromide (CTAB) method [72]. DNA was quantified by a Nanodrop 2000 UV-vis spectrophotometer machine and diluted to 50 ng μl^−1^ with OD260/280 of 1.8–2.2. RNase A (ST579, Beyotime, China) was used to remove RNA contamination.

### Classical mapping markers

After integrated linked markers of *ari-e* from previous mapping reports [19, 26, 64, 66] to the barley consensus map of chromosome 5H [48], we estimated that the genetic position from 45 cM to 60 cM as the target region of the *ari-e* gene. The 29 InDel markers in the *ari-e* gene region reported by Zhou et al. [48] were used to detect polymorphism between Dash and VB9104. Based on the current barley assembly information, *Hvces8* (*MLOC_68431*) was identified to the target region and was sequenced to find polymorphism between Dash and VB9104. According to Liu et al. [26], Morex_contig47526 was most closely associated with *ari-e*. So it was screened for microsatellite DNA; two pairs of potential SSR markers were designed using Oligo Primer Analysis Software v.7. PCR amplified DNA from the parental lines and DH individuals as a template. The final volume was set to 20 μl containing 1 × Taq Mix (GST101, Bioteke, China), 0.3 μM of each primer and 200 ng of template DNA. The PCR reactions were performed as follows: denaturation at 94 °C for 3 min, followed by 35 cycles of 94 °C for 30 s, 60 °C for 30 s, 72 °C for 30 s, and a final extension at 72 °C for 5 min. PCR products were separated on 6 % polyacrylamide gel (acrylamide/bisacrylamide ratio of 37.5:1) in 0.5 × Tris-borate-ethylene-diaminetetra-acetic acid and run at room temperature for 2–4 h, stained with silver nitrate, and observed on white illumination. Size differences in polymorphisms were identified between Dash and VB9104. Primer sequences and amplified lengths of the polymorphism markers used in this study are listed in Table 1.

### Construction of SLAF library for sequencing and analysis of SLAF-seq data

We selected 30 progeny of the Dash × VB9104 DH population with the lowest and highest stature and bulked their DNAs to make ‘semi-dwarf’ and ‘high’ pools, respectively. The parents and two pools were used for SLAF library construction and sequencing as described previously [49]. DNA was digested with the restriction enzyme *Rsa*I. Paired-end sequencing with a read length of 100 bp was conducted on the platform of Illumina Genome Analyzer II (Illumina Inc., San Diego, CA, USA). After sequencing, all reads were aligned to the reference sequence released by The International Barley Sequencing Consortium (IBSC) using BWA software [73]. SNP calling was performed using GATK software (https://www.broadinstitute.org/gatk/guide/best-practices.php). We excluded SNPs which supported less than four reads in the two pools and showed no polymorphism between the parents because they may be false positives due to genomic repeat sequence, sequencing or alignment errors. Association mapping was conducted to identify candidate regions for plant height using the SNP-index (see [74, 75] for methods). The Δ(SNP-index) was obtained by subtracting the SNP-index of the semi-dwarf pool from that of the high pool. For each read depth, 99 % confidence intervals of Δ(SNP-index) were obtained following Takagi et al. [75]. Default parameters were used with all software.

### Markers development by SLAF-seq strategy and Dash × VB9104 DH population genotyping

To minimize the genetic interval for fine-mapping and to verify the accuracy of SLAF-seq, SNP flanking primers located in the traditional mapping position of *ari-e* in the Dash × VB9104 DH population were designed using Oligo Primer Analysis Software v.7 which ranged from 100 to 300 bp in length. PCR amplification was conducted as described above. Amplicons were sequenced in one direction using the specific PCR primers distal to the potential SNP position by Shanghai Sunny Biotechnology Co., Ltd. The Megalign program (DNAStar) was used for sequence alignment and to confirm SNP or InDel sites. The confirmed markers were genotyped in 119 Dash × VB9104 DH individuals following SNP marker detection with direct DNA sequencing.

### InDel markers developed using the whole-genome shotgun strategy

In this study, the whole-genome shotgun strategy was selected to perform resequencing of the barley cultivar Hindmarsh with *ari-e*. A paired-end library with insertion size of about 350 bp was prepared and sequenced on the platform of Illumina HiSeqTM2000 in BGI-Shenzhen. After the raw reads had been produced, strict filtering was performed to ensure the reliability of further analysis by removing contaminated or low-quality reads. Clean reads were mapped to the reference genome sequence using the publicly-accepted aligner of BWA with default parameters. The reference used for reads mapping was population sequencing methodology (POPSEQ) [64]. PCR duplication was removed using rmdup in the SAMtools software package. After alignment, only reads with a mapping quality score >40 were used to infer SNP and InDel variations using the classic pipeline of mpileup/bcftools in SAMtools. In this step, variations with less than five supporting reads or those located within 10 bp around both ends were removed. At the same time, the genetic effect was assessed for these variations using SnpEffsoftware based on their gene structure and locations. Moreover, the CDS sequences of these genes containing significant mutations were used to search homology proteins in databases of NR, Swiss-Prot, KEGG, COG and GO for gene function and involved pathway prediction.

### Markers validation on the Hindmarsh × W1 DH population

Five markers showing polymorphism between Hindmarsh and W1 were used to genotype 340 individuals from the Hindmarsh × W1 DH population. PCR and genotyping methods are as described above.

### Genetic mapping

Linkage analysis was conducted using MAPMAKER version 3.0 software [76]. Map distances were estimated using the Kosambi equation [77].

### Real-time quantitative RT-PCR of the predicted genes

RNA was extracted from the leaves of VB9104, Dash and Golden Promise collected at the heading stage using a Spin Column Plant Total RNA Purification Kit (SK1321, Sanggon Biotech (Shanghai) Co., Ltd). cDNA was prepared from 1 μg RNA using an AMV First Strand cDNA Synthesis Kit (SK2445, Sanggon Biotech (Shanghai) Co., Ltd). qPCR reactions were performed using SYBR Green (B639273, SG Fast qPCR Master Mix (High Rox), BBI) and the Applied Biosystems Step-one plus Real-time PCR System. The real-time PCR assays were performed in triplicate for each cDNA sample. To determine the transcription levels of the five genes in the candidate region, *HvACTIN* and *HvGAPDH* were employed as reference genes. Additional file 2: Table S2 lists the oligonucleotide sequences used for quantitative RT-PCR. For statistical analysis of qPCR data, cycle threshold (C_T_) values were used to determine *∆* C_T_ values (*∆* C_T_ = C_Ttarget_ –C_Treference_), and expression levels of target genes relative to reference genes were determined as 2^-*∆* CT^.

## Additional files

Additional file 1:
**Table S1.** Genetic variants of the target region. (XLS 34 kb)
Additional file 2:
**Table S2.** Oligonucleotide sequences used in qRT-PCR assays. (XLS 18 kb)
Additional file 3:
**Figure S1.** Relative expression levels of five predicted genes. (DOC 38 kb)

## Abbreviations

BSA: Bulked segregant analysis
DH: Doubled haploid
GA: Gibberellic acid
GBS: Genotyping by sequencing
GPMx: Golden Promise × Morex
IBSC: International Barley Sequencing Consortium
NGS: Next-generation sequencing
PAGE: Polyacrylamide gel electrophoresis
PCR: Polymerase chain reaction
POPSEQ: Population sequencing
QTL: Quantitative trait locus
RRL: Reduced representation library
SLAF-seq: Specific-length amplified fragment sequencing
