# Supplementary material for: Marker development using SLAF-seq and whole-genome shotgun strategy to fine-map the semi-dwarf gene ari-e in barley
Source: BMC Genomics. 2016 Nov 11;17:911. doi: 10.1186/s12864-016-3247-4 (PMC5106812; doi:10.1186/s12864-016-3247-4)
Supplement: Additional file 3: — Figure S1. Relative expression levels of five predicted genes. (DOC 38 kb) [file 12864_2016_3247_MOESM3_ESM.doc]

Figure S1. Relative expression levels of five predicted genes.

Relative expression level

*HvLOC347149*

Relative expression level

*MLOC_15960*

Relative expression level

*MLOC_66038*

Relative expression level

*MLOC_72534*

Relative expression level

*HvLOC100842002*
